# Clinical Characteristics, Outcomes, and Cost Associated with Inpatient Intensive Care for Infectious Keratitis

**DOI:** 10.3390/medicina61091680

**Published:** 2025-09-16

**Authors:** Anne Strong Caldwell, Ari M. Stoner, Ellen Rhodes, Nihaal Mehta, Michael C. Chen

**Affiliations:** Department of Ophthalmology, University of Colorado School of Medicine, Aurora, CO 80045, USA

**Keywords:** infectious keratitis, corneal ulcer, healthcare costs, social determinants of health

## Abstract

*Background and Objectives*: Infectious keratitis (IK) is typically managed in an outpatient setting, but patients with severe infections or significant social barriers may require hospital admission. In safety-net hospital systems, these admissions to the intensive care unit (ICU) occur due to hospital protocols for frequent topical antibiotic administration. This study aims to characterize the ocular and social risk factors, visual outcomes, and financial costs associated with ICU admission for IK in an underserved population. *Materials and Methods*: We conducted a retrospective case series of all patients admitted to the ICU for primary treatment of IK at the Denver Health Medical Center between 1 January 2017 and 31 December 2022. Patients admitted for other medical issues with concurrent IK were excluded. Demographic data, ocular and social risk factors, microbiological culture results, reasons for admission, length of stay, hospital charges, and clinical outcomes were obtained via chart review. *Results*: Fourteen patients with 16 ICU admissions were included. The average age was 51.7 years, and 79% were male. Most patients endorsed current illicit drug use (71%), and 36% were unhoused. The most common ocular risk factor was trauma (43%). Mean length of stay was 7.43 days, with a mean hospital charge of $48,535.90 per admission. Most ulcers were large (88%) and presented with poor vision (only 19% had better than hand motion vision). The most common reason for ICU admission was concern about outpatient compliance (63%). At last follow-up, 40% of patients had stable vision and 40% had improved vision compared to admission. *Conclusions*: ICU admission for IK in patients with significant social barriers may preserve vision, but it comes with substantial financial and societal cost. Alternative care strategies and preventative interventions should be considered to reduce reliance on ICU resources while maintaining effective treatment.

## 1. Introduction

Infectious keratitis (IK), the fifth leading cause of blindness worldwide, is a painful vision-threatening condition requiring intensive treatment to prevent irreversible vision or globe loss [1]. IK is an infection of the cornea, also known as an infectious corneal ulcer, which can be caused by bacteria, fungi, parasites, or viruses. Infectious keratitis is usually preceded by an insult to the cornea by way of trauma, contact lens use, or an aggressive penetrative infection wherein microorganisms penetrate the corneal epithelium and stroma. This results in an infiltrate which can generate a potent inflammatory response that is both painful and vision threatening. In the United States, about 1 million visits to health professionals are attributable to IK, with 58,000 visits to emergency departments, and has been estimated to cost around USD 175 million annually to the US healthcare system [2].

The preferred treatment for severe cases of IK consists of two fortified topical antibiotics for broad coverage with a loading dose of drops every 5 to 15 min followed by an hourly instillation of drops [3]. Frequent instillation of drops, as well as use of the loading dose, is rationalized as antibiotics have poor corneal stroma penetration, and the minimum inhibitory concentration of the antibiotic must be reached in the stroma for therapeutic effect. While IK is typically managed in an outpatient setting in the United States, patients with IK and significant barriers to adequate and consistent therapy often require admission for inpatient care.

Relatively little research has been performed in the US analyzing hospitalization rates associated with IK treatment, with most of the contemporary studies focusing on data from the National Inpatient Sample (NIS) [4,5]. Lee et al. found that from 2002 to 2012, an estimated 19,878 patients were seen in the emergency department for IK, with an estimated 4.2% (850) requiring admission to the hospital for treatment of their IK [4]. More recently, Akosman et al. published a study using data from the NIS between 2015 and 2020, and found that patients who were Black, older than 55 years, experiencing housing insecurity, or insured by Medicare were more likely to have longer hospital lengths of stay (LOS) [5]. Additionally, they presented on average total charges that were incurred from the hospital stays, and found that the average charge for hospital LOS ≤ 4 days was $26,474, while the average charge for LOS > 4 days was $79,504 [5].

At Denver Health Medical Center (DHMC), a safety-net hospital system that cares for a high proportion of un- or under-insured patients, when a patient is admitted to the hospital for treatment of IK, hospital policy requires that the patient be admitted to the ICU due to the frequency of eye drop instillation. To the best of our knowledge, no published data exists regarding the practice of ICU admission for severe IK, but this practice is in place at other institutions and anecdotally seems to be a widespread pattern within ophthalmology. These hospital stays are associated with high financial cost as well as allocation of an ICU bed to a typically otherwise medically stable patient. The objective of this retrospective chart review is to examine the clinical characteristics, social risk factors, individual reason for admission, outcomes, and cost of the hospital stay in patients requiring hospitalization for IK at DHMC between 2017 and 2022. While previous studies on hospitalization for IK were performed on NIS epidemiological data [2,3], this study adds to the existing literature with descriptive patient-level data on the reasons for each individual hospitalization and outcomes.

## 2. Materials and Methods

All patients admitted to the DHMC hospital for primary treatment of IK during the five-year period of 1 January 2017 and 31 December 2022 were included in this retrospective case series. Any patients admitted for an alternate medical problem with concurrent treatment of IK were excluded from this study. All infectious causes of IK were included in our analysis. This study was determined to be exempt from review by the Colorado Multiple Institutional Review Board and was conducted in compliance with the Declaration of Helsinki.

Each patient chart was reviewed by an ophthalmologist, and a database was created with information on demographics, ocular risk factors associated with IK, treatment, reason for admission, social risk factors, presenting and final visual acuity (VA), organism identified by culture, and size of ulcer at admission. Regarding general demographics, the patient’s sex and race/ethnicity noted in the electronic health record were recorded. Race and ethnicity were collected for this study as prior reports have found significant associations between race and longer LOS in the hospital for patients admitted for treatment of IK [5]. We determined the ocular risk factors for IK in our cohort based off prior studies which showed that eyelid malposition, contact lens use, ocular trauma, surgery within 1 month of developing IK, history of herpes simplex virus (HSV) or varicella zoster virus (VZV) keratitis, and history of dry eye syndrome were significant risk factors for IK [6,7,8,9]. The ulcer was classified as small if <1 mm, medium if 1–1.5 mm, and large if >1.5 mm [10]. We determined social risk factors for IK based off prior studies that showed that being unhoused or engaging in illicit drug or alcohol use were correlated with longer LOS for treatment of IK [5]. In addition to ocular and social characteristics, financial data including the total hospital charges and outstanding patient balances were obtained via chart review.

Reasons for admission included compliance concern and demonstrated non-compliance outpatient. Compliance concern was defined as the concern that the patient would be unable to administer the drops at the necessary frequency outpatient, while demonstrated non-compliance outpatient was defined as a patient who initially was treated on an outpatient basis but demonstrated inability to administer their drops at the necessary frequency. Other reasons for admission included patients that were previously lost to follow-up (LTFU) while outpatient, patients at risk of perforation, cost of drops, and the correctional facility being unable to administer drops at the necessary frequency. Descriptive statistics are reported as frequency and percentage.

## 3. Results

After exclusion criteria was applied, 14 patients and 16 admissions were included for the analysis. The patient demographics are shown in Table 1 and clinical characteristics of each admission are shown in Table 2. The average LOS in the ICU was 7.43 days ± 5.87 (range: 2–25 days) with a mean total hospital charge for each stay of USD 48,535.90 (SD USD 36,461.59, with a range of USD 15,770.90–USD 160,639.56).

Upon admission, most cases were treated with fortified vancomycin (25 mg/mL) and tobramycin (15 mg/mL), alternating every 30 min (81%). One patient (6%) was treated with fortified vancomycin and tobramycin, alternating every hour, one patient (6%) was treated with fortified vancomycin and moxifloxacin (0.5%) every 30 min, and one patient (6%) was treated with fortified vancomycin, tobramycin, and amphotericin B (0.15%), alternating every 30 min. Antibacterial treatment was augmented with antivirals (valacyclovir, 1 g, three times a day, or acyclovir, 400 mg, five times a day) in four patients (29%), and seven (50%) patients additionally received a high dose of vitamin C (1 g daily) and doxycycline (100 mg twice daily).

All patients had multiple reasons for admission to the hospital (Table 3). Concern about compliance in an outpatient setting was the most common reason for admission (63%). Of the patients who were admitted to the ICU twice (*n* = 2), one was re-admitted due to being LTFU outpatient with worsening of the ulcer, while the other was re-admitted due to incarceration and the correctional facility being unable to administer drops at the necessary frequency.

Most patients were seen at least once at DHMC after leaving the hospital (64%), 2 patients (14%) followed up at outside clinics, and 2 patients (14%) were LTFU. The final VA documented in the chart, either at discharge, or at follow-up, found that only 4 (40%) had VA of 20/200 or better. Four patients (40%) had improved VA compared to their admission VA, and four patients (40%) had stable VA from admission. There were two corneal perforations: one patient received a penetrating keratoplasty (PKP), and the other patient developed panophthalmitis and underwent enucleation. Two patients received a tarsorrhaphy, one of whom went home on hospice, and the other was LTFU. Finally, 10 patients had resolution of IK with formation of a scar. Most charts did not document why a PKP was not performed after resolution of the infection, but two charts noted that a PKP could be considered when the patient was no longer incarcerated, and one chart noted that the patient was not a good candidate for PKP due to corneal hypoesthesia.

## 4. Discussion

To the best of our knowledge, this is the first study of a contemporary US cohort that provides patient-level data on reason for admission, social and clinical risk factors, outpatient outcomes, and hospital charges for patients with IK that required ICU admission for care.

In our cohort, the primary reason for admission included concern about treatment regimen compliance as expressed by the patient, followed by a demonstrated lack of compliance in an outpatient setting. In addition to these social reasons for admission, most of our patients had large ulcers and poor vision at admission, which in and of themselves are reasonable indications for admission. Other studies performed outside the US have cited severe keratitis as the reason for admission. However, in each of these papers, it is unclear how severe keratitis was defined; there was no discussion of hospital or national policies for treatment of IK, and social reasons for admission were not discussed [6,7]. In US studies performed on the NIS database, results include information on social, demographic, and health risk factors associated with admission. However, these epidemiological studies lack patient-level data on social reasons for admission and outcomes of outpatient follow-up [4,5]. Furthermore, neither study comments on whether the patients were admitted to the ICU or general hospital wards [4,5].

Similarly to the NIS studies, public insurance (Medicare or Medicaid), housing insecurity, and drug or alcohol use were common in our cohort [4,5]. Our cohort’s average LOS and clinical risk factors associated with the development of IK were also similar to findings from prior studies [4,5,6,7,8,9]. Most ulcers did not perforate, and at last follow-up, most patients had stable or improved vision. While it is unknown how our patients would have fared if treated in an outpatient setting, assuming that drop compliance would have been the primary issue without admission, it can be argued that these admissions resulted in preserved or improved vision and prevented complications such as perforation or endophthalmitis in the majority of our cohort.

While these hospital admissions might have been vision- or globe-saving, the significant cost associated with admission to the ICU for an otherwise medically stable patient should be discussed, and alternate options to provide care at decreased cost should be considered. Few other contemporary studies comment on the cost of hospital stays for inpatient treatment of IK, and ours is the first non-epidemiological study to discuss the financial burden associated with inpatient treatment of IK. The mean total hospital charge for our cohort was USD 48,535.90, similar to NIS based estimates of USD 21,034.04 for Lee’s cohort which included patients from 2002 to 2012, and USD 52,809 for Akosman’s cohort which included patients from 2015 to 2020 [4,5]. Based on the mean total hospital charge, Akosman et al. estimated a direct annual healthcare expenditure of USD 35,819,590 for treatment of inpatient IK [5]. Unfortunately, the NIS-based studies do not differentiate between ICU vs. general wards admission. However, our financial data, in combination with NIS epidemiological cost estimates, highlight the need to consider ways to decrease financial burden associated with hospital stays for IK.

In addition to the financial burden, there is also a considerable societal cost of utilizing an ICU bed, nurse, physician team, and support staff—a system intended for managing life-threatening conditions—for an otherwise medically stable patient without any risk to life. Avoiding admissions to the ICU for treatment of IK would result in decreased costs as well as lower ICU bed utilization. According to the Hospital Price Index estimator for DHMC, in 2024 the estimated average charge of a medical ICU bed is USD 8754.89, while the estimated average charge of a general wards bed is USD 2361.40 [11]. As such, finding solutions to avoid ICU admission is an important starting point for driving down both financial and societal costs associated with these hospital stays and freeing an ICU bed for a patient with a life threatening condition.

While the frequency of treatment for patients with IK is seemingly high (hourly to every half-hour drops), the actual task of instilling the drop is fast, easy, and low risk. Unfortunately, because eye drops are considered a medication equivalent to other systemic medications, many states require administration by registered nurses (RNs) only. Certain states, including Colorado, allow certified nursing assistants (CNAs) to pursue further training in medication administration. These licensed CNA-Medication Aides (CMA) can administer and record certain ophthalmic, oral, nasal, and otic medications [12]. Some states only allow CMA staffing in nursing home settings; however, in states that allow hospital-based CMAs, an ICU-level admission for frequent RN-level intervention could be avoided.

Finally, another potentially viable strategy to avoid admission to the ICU is employing subconjunctival antibiotics (SCAs). While SCAs are not commonly used for treatment of IK, the World Health Organization and American Academy of Ophthalmology Preferred Practice Patterns discuss consideration of SCAs as a possibility if compliance is a concern [3,13]. If SCAs were employed, the patient’s treatment could be augmented with additional antibiotic drops, but at a lower frequency, thus avoiding admission to the ICU. Several papers found that in a comparison of subconjunctival cefazolin or gentamicin versus drops in an animal model, there was no difference in bacterial counts at their endpoints [14,15,16]. Unfortunately, results from other reports have shown that drops were more efficacious in eliminating bacteria than periocular injection [17,18]. Human studies on SCAs have been limited to case reports with several cases demonstrating shortened treatment duration with use of subconjunctival amphotericin B or fluconazole as adjuvants to topical and systemic therapies for treatment of fungal keratitis [19,20,21]. SCAs do carry unique risks, including conjunctival necrosis following amphotericin B, hemorrhagic occlusive vasculitis following inadvertent intraocular injection of gentamicin, corneal opacification after injection of soframycin, and globe perforation [22,23,24]. The decision to manage IK with SCAs would require consideration of the frequency of the injection and topical drop administration, and close monitoring of the patient given the lack of studies in human subjects. Clinical trial data on use of SCAs in humans for IK could further guide the feasibility of this strategy.

While attempts to decrease the cost of inpatient admission for treatment of IK must be considered, implementing healthcare protocols to avoid delayed presentation with advanced large ulcers and poor VA is the ultimate way to save vision and decrease cost. In a recent commentary, Prajna et al. discuss public health strategies aimed to treat IK at an earlier stage, including starting antibiotics as soon as a patient sustains a corneal abrasion, using tele-ophthalmology to increase accessibility to patients, and employing point of care microbiological diagnostic kits to guide treatment [25]. By treating IK in the early stages, it is possible the vision threatening complications and significant cost of treatment could be decreased.

Limitations to this study include the retrospective nature, small sample size, and reliance on the medical chart for review. While all patients had stated reasons for admission written in the chart, it is difficult to know the extent and details of each patient’s social situation that prompted admission. Furthermore, our study is observational and lacks a comparison arm. Given the patient population is from a safety-net hospital system, it is possible that our findings are not generalizable. However, we believe the patient-level characteristics and charge data presented in this study add to the growing literature highlighting the global burden of IK.

## 5. Conclusions

Based on our study results, we believe it is worthwhile for any patient with potentially visually threatening IK who is identified as having significant barriers, particularly socioeconomic, to be admitted to the hospital. We recommend that each hospital system take an individualized approach to devise a strategy to mitigate the financial, staffing, and institutional burden associated with these admissions. Further studies are needed on potential alternative therapeutic options, such as SCAs or early therapeutic interventions, that may obviate the need for costly admissions for IK treatment without negatively impacting patient outcomes.

## Figures and Tables

**Table 1:** Patient Demographics.

Characteristic	*n*	%
**Sex**		
Male	11	79%
Female	3	21%
**Race/Ethnicity**		
Hispanic	6	43%
Non-Hispanic White	7	50%
Unknown	1	7%
**Language**		
English	13	93%
Spanish	1	7%
**Primary Insurance**		
Medicaid	8	57%
Medicare	2	14%
Medicare + Medicaid	1	7%
Uninsured	2	14%
Colorado Department of Corrections	1	7%
**Repeated Subjects**		
Number of Patients Admitted Twice	2	-
**Social Risk Factors**		
Unhoused	5	36%
Illicit Drug Use	10	71%
Alcohol Use	4	29%
**Mean ± Standard Deviation**		
Age	51.7	±15.29
Length of Stay in ICU (Days)	7.43	±5.87

**Table 2:** Clinical Characteristics.

Characteristic	*n*	%
**Eye**		
Right	6	43%
Left	8	57%
**Visual Acuity at Presentation**		
≥20/200	2	13%
Count Fingers	1	6%
Hand Motion	8	50%
Light Perception	4	25%
No Light Perception	1	6%
**Size of Ulcer at Presentation**		
Medium	1	6%
Large	14	88%
Unknown	1	6%
**Bacteria Isolated by Culture**		
Coagulase-Negative Staphylococci	3	19%
Group B Streptococcus	1	6%
Corynebacterium	1	6%
Moraxella	3	19%
Polymicrobial	5	31%
No growth	3	19%
**Ocular Risk Factors ^1^**		
Eyelid Malposition	2	14%
Contact Lens Use	2	14%
Ocular Trauma Prior to Ulcer	6	43%
History of ocular HSV/VZV	2	14%

^1^ Excluded re-admissions from count.

**Table 3:** Reason for admission (*n* = 16).

	*n*	%
Compliance Concern	10	63%
Demonstrated Non-Compliance Outpatient	7	44%
Lost to Follow-Up Outpatient	2	13%
Risk of Perforation	3	19%
Correctional Facility Unable to Administer Drops	2	13%
Cost of Drops	1	6%

## Data Availability

The data presented in this study are available on request from the corresponding author.

## Abbreviations

The following abbreviations are used in this manuscript:

IK	Infectious Keratitis
ICU	Intensive Care Unit
NIS	National Inpatient Sample
LOS	Length of Stay
DHMC	Denver Health Medical Center
VA	Visual Acuity
VZV	Varicella Zoster Virus
HSV	Herpes Simplex Virus
LTFU	Loss To Follow-Up
CNA	Certified Nursing Aide
CMA	CNA-Medication Aide
SCAs	Subconjunctival Antibiotics
